# Severe mutilating injuries with complex macroamputations of the upper extremity – is it worth the effort?

**DOI:** 10.1186/s13017-015-0025-6

**Published:** 2015-07-07

**Authors:** Katrin Stanger, Raymund E. Horch, Adrian Dragu

**Affiliations:** Department of Plastic and Hand Surgery, OKM Orthopädische Klinik Markgröningen gGmbH, Markgröningen, Germany; Department of Plastic and Hand Surgery, University Hospital of Erlangen, Friedrich-Alexander-University Erlangen-Nürnberg, Erlangen, Germany; Department of Plastic and Hand Surgery, Klinikum St. Georg gGmbH, Leipzig, Germany

**Keywords:** Complex macroamputation, Avulsion, Replantation, Multiple injuries, Upper extremity, Functional outcome

## Abstract

**Introduction:**

An amputation of the upper extremity and the following replantation is still one of the most challenging operations in the field of reconstructive surgery, especially in extremely severe cases of combined mutilating macroamputations including avulsion and multilevel injuries. Specialists agree that macroamputations with sharp wound edges are an absolute indication for replantation. However, there is no agreement in disastrous cases including avulsion and multilevel injuries. The outcome of the operation is depending on several factors, including the type of accident, age and pre-existing disease of the patient, as well as time of ischemia and appropriate physical therapy.

**Methods:**

Between January 1^st^ 2003 and December 31^st^ 2011 six patients underwent a macroreplantation with disastrous combined and complex injuries of the upper extremity in our department. We performed a follow up and evaluated the functional outcome of the upper extremity function using the DASH questionnaire (average follow up of 3.1 years).

**Results:**

The mean time of ischemia was 04:50 h (02:46 h–06:17 h). The mean time for the operation was 05:30 h (01:55 h–08:20 h). The mean operations needed per patient were 7 (2–16). The average hospital stay was 29d (16–59d). According to the DASH-Score from five out of six patients the functional outcome of the replanted extremity has a mean score of 71 points. The versatility of the replanted extremity in the field of work had 95, and sport, music was assessed with a mean score of 96 points.

**Conclusions:**

Severe and disastrous combined and complex macroamputations of the upper extremity may also have an absolute indication for replantation even though the functional outcome is poor. Not only the feeling of physical integrity can be restored, but the replantation of an amputated upper extremity enables complete or partial recovery of function and sensibility of the arm which is important for the individual. Although our results show a very high DASH-Score, those achievements justify time and person consuming operations. In most cases a replanted extremity is still superior to a secondary allotransplantation. Usually the use of prosthesis is not favored by the treated patients.

## Introduction

In 1914 the Austrian Ernst Jeger performed the first successful replantation of an amputated upper arm [1]. The two authors Malt and McKhann have operated successfully the first replantation of a hand with a 12 year old in 1962 [2].

Amputations of the upper extremity proximal to the radiocarpal joint or the ankle are referred to as macroamputations. Distal to these regions the amputations are specified as microamputation [3]. A macroamputation injury should be treated initially as a patient with a polytrauma [4]. An amputation of the upper extremity can be found in 0.2–3.0 % in a polytraumatized patient. In this context it is very important to differentiate between macroamputations with sharp wound edges and disastrous and severe complex cases including avulsion or multilevel injuries. The ischemic tolerance of the amputated part of the body depends on the content of muscle. An amputated finger tolerates a time of ischemia of up to 24 h, while a macroamputat containing a mass of muscles should not exceed a time of ischemia of more than eight hours [5].

The successful treatment of macroamputations of the upper extremity requires an expert knowledge and operating experience in the field of microsurgery [6–12]. Overall, the incidence of amputation injuries is declining due to modern safety precautions. Most accidents are industrial accidents, of which mainly work on machines with cutting surfaces and rollings can be classified as dangerous. An amputation of the upper limb results for the affected person in a significant functional, aesthetic and psychological impairment [7, 13]. Despite the impressive technical refinements of the artificial limbs the removable denture can only partially compensate for the functional limitations [13, 14]. Furthermore still the mental trauma remains due to the missing body integrity [13]. The ideal goal of revascularization or replantation of the upper extremity with the help of modern microsurgery is regaining a sensitive limb containing a protective sensibility with motorized gripping, holding and supporting function [7, 8, 13]. Due to a significantly better quality of life of these patients the indication for replantation is absolute, even with regard to higher costs, additional effort through serveral operations as well as longer postoperative care and non-productive time after reconstruction [13]. According to the current literature in up to 82 % of the affected patients a functional limb can be recuperated in macroamputations without avulsion and additional disastrous combined injuries.

The indication in favour or against the preservation of the affected upper extremity must take into account the individual circumstances of the patient and the case itself [15, 16]. As an additional support for making the decision the use of the mangled extremity scoring system “Ganga Hospital Score” first described by Shanmuganathan et al. and Rajasekaran et al. may be helpful [17, 18]. In addition the new OTA (Orthopaedic Trauma Association) open fracture classification may be of additional help [19]. On the other hand other scoring systems such as MESS (“mangled extremity scoring system”) do not include any prediction regarding the functional outcome of replantation and therefore are not a validated scoring system for guidance of ampuations versus limb salvage in critical extremity injuries. “Life before limb” of course always remains a matter of principle [14, 20–22].

In case of life-threatening associated injuries of patients the replantation should be avoided or in appropriate cases (amputation of the hand or lower extremity) an interim solution can be discussed such as vascular connection to the axilla or the groin. As soon as the patient is stable enough a secondary replantation to the extremity is possible [21].

The success rate after replantation or revascularization depends on the extent of damage to the amputat, as well as the level of amputation, the soft tissue damage of the amputat and of the stump, the type and duration of ischemia, as well of the patient’s age [6–8, 13, 23]. The level of amputation and the kind of trauma, as well as the patient’s age seem to have the most important influences on the quality of the outcome (Table 1) [13]. All off our presented cases are mutilating severe and complex macroamputations with additional avulsion or multilevel injuries.

**Table 1:** Influencing factors for the functional outcomes of macroreplantations

Dependent relations of the functional outcome after macroreplantation of the upper extremity	
Therapy	-First aid at accident location
	-Transport to the hospital
	-Time of ischemia
	-Experience of the replanting team
Patient	-Diseases
	-Age
	-Compliance
	-Motivation
Amputat	-Level of amputation
	-Damage of the amputat (avulsion and/or multillevel injury)
	-Mechanism of trauma

In the literature, the overall success rate of macroreplantationen is indicated with 76–100 % for total amputation, and indicated with 89.7 % for subtotal amputations [3, 4, 11]. Depending on the type of amputation injury the success rate for an even cutting damange injury averages 81.9 %, for localized crush injury it is 79.3 %, for amputation injuries with diffuse bruising it adds up to 87 % and for avulsion injuries it averages 68.0 % [11, 13]. With nowadays advanced strategies for replantations and microsurgical techniques for macroreplantation the exclusive claim for a viable and revascularized extremity may no longer prevail. Aspects such as functionality, aesthetics, painlessness and integration of the replanted limb into the overall picture of the patient and his profession need to be determining factors for the surgeon in deciding for the operation [9, 10, 24].

While in amputations of fingers the surgeon distinguishes between relative and absolute indications for replantation, in cases of macroamputations there is common agreement for an absolute indication for revascularization/replantation, as long as no relevant contraindications exist [21]. A relevant contraindication for example would be an unstable patient who would not survive a prolonged operation such is previewed for a replantation. Among others a warm ischemia over 12 h and a cold ischemia over 24 h, as well as a complex multi-level injury with massive destruction of amputated limb counts as a contraindications for replantation [10, 21, 22]. Some authors propagate, that only the amputated limb, which has the prospect of a successful revascularization, and obtaining a partial function of the limb should be chosen to operate on [12]. These authors say that a loose-hanging, functionless replanted limb is of no use to the patient and may lead to secondary request for amputation [7]. On the other side a subjective evaluation of results after replantation shows a high degree of bonding between the patient and his reconstructed limb no matter how the functional outcome is [25].

For the upper limb, in contrast to the lower limb the secondary amputation is an absolute exception [13]. The review of the literature reports an incidence of 2.5–8 % of affected patients. Obviously the artificial replacement of the upper limb with a prosthesis is only tolerated in only 20 to maximum 50 % of the cases. A situation of chronic pain is reported to occur in 5–10 % of the patients [13].

### Technical aspects

#### Intraoperative management

One of the most important operational steps is the primary debridement of necrotic tissue [14, 8, 23]. This includes in the “zone of injury” not only the radical removal of foreign bodies and contaminations, but also involves the debridement of non-vital muscle, as well as the shortening of the fractured bone in the area of injury [7]. This is eminent to avoid postoperative infections caused by non-vital and necrotic structures. After debridement the decision for vascular, nervous or tendon grafts for a tension-free reconstruction can be determined. A stable and definitive bone fixation is desirable in primary care [21]. Macroamputations should primarily be fixed with plates and screws. An external fixation is advisable in complicated interjoint bony fractures with extensive debris zone [7]. After the treatment of the bone the tendon suture, the arterial and venous vascular suture and at last the nerve suture stands in line.

Due to the massive swelling of the almost always expected reperfusion injury and possible expansion of the demarcation zone, a complex soft tissue reconstruction as primary treatment is not indicated in most cases. Therefore a temporary coverage of the soft tissue with a negative pressure system is highly recommended. It enables further debridements in case of progressing necrosis as well as the final secondary soft tissue coverage under detumescent conditions later on [21, 23]. An initial adequate bony debridement with appropriate shortening of the bone may avoid the use of vascular, nervous or tendon grafts. In any case, the primary treatment is crucial for a good functional result. If tension-free sutures are not possible, the indication for a graft should always be made generously. The target is to reconstruct two veins for each reconstructed artery [7]. The compartments of the upper limb, the carpal tunnel as well as the Loge de Guyon should be split in order to avoid a postoperative compartment syndrome [26]. The two most common reasons for the failure of a replantation are the muscle necrosis and subsequent an infection, both can lead to a postoperative increasing swelling and thrombosis of the reconstructed vessels. It is therefore advisable to schedule the patient for a second look 48–72 h postoperatively in order to evaluate the viability of the soft tissues. An amputation of the upper extremity can lead to massive blood loss. The necessity for blood transfusion should be recognized timely [9].

#### Postoperative management

Ensuring a successful replantation depends on a stringent postoperative management [7]. After surgery, the hand has to be checked every hour for 72 h. The color of the skin of replanted extremity, the turgor, the vascular refill and temperature are crucial criteria. A closure of the arteries or veins, caused by a thrombus or due to severe swelling (compartment syndrome) should be diagnosed in time for early revision. A compartment syndrome, including swelling of the soft tissues or a hematoma may induce an increase of pressure, which can lead to hypoperfusion of the replanted extremity. Already the suspicion for a compartment syndrome should alert the surgeon to indicate an emergency revision.

An axillary plexus is ideally provided for the patient right from the beginning. The early sympatholysis is recommended for postoperative pain therapy and as well as a prophylaxe for developing an algodystrophy. The limb should be put up on a soft surface to reduce the swelling. Immobilization is achieved by a splint, which should be very loose at the beginning to avoid any pressure sores.

The level of hemoglobin should be monitored daily during the first postoperative days. A pre- and intraoperative blood loss can make a blood transfusion indispensable. A rapid drop of the hemoglobin may indicate the formation of a hematoma. Likewise the parameters myoglobin and creatine kinase should be monitored daily. Through the trauma and surgery large parts of the muscle are often bruised and injured, so the level of myoglobin may be increased. The clinical image of a crush kidney is likely to occur, which can lead to acute renal failure [7, 21]. Therefore the patient should be sufficiently provided with water in order to flush the kidneys. A urinary catheter is recommended to monitor the urine concentration and the excretion. Right from the beginning as well as in the medium and long-term postoperative follow-up an interdisciplinary psychosomatic should be initiated. The loss of a large body limbs is not only a significant turning point in the perception of the body, but also in the mental status. For the long term success of a replantation a well trained and experienced team is essential. Trained nursing staff, physiotherapists and occupational therapists, pain specialists and psychiatrists, as well as an experienced orthopaedic technicians should be part of this team. The rehabilitation is long, time consuming, costly and stressful [10, 27]. The patients should be attached to the clinic for many years. A regular follow-up by all members of the team, usually over years, is extremely important for the rehabilitation of the extremity into the overall picture of the patient and into the everyday life and work. Follow-up operations like arthrodesis, resection of a pseudarthrosis, a tenolysis or a nerve or tendon reconstruction may be necessary. Often the removal of metal, an improvement of a scar, a resection of a neuroma or a motoric reconstruction is required [7].

The affected patients should be informed that the replanted limb rarely achieves fully pretraumatic function. However, the possible achievable results are superior to any stump with subsequent prosthetic fitting or even secondary orthotropic transplantation with long life immunosuppressive therapy.

The functional outcome of a replantation depends on the level of amputation, the type of injury, the duration of ischemia, the sex and age of the patient, concomitant diseases, as well as on the existing noxious substances such as nicotine and alcohol consumption and the motivation of the patient. In particular the age of the patient appears to be of great importance for the possible functional outcome [6, 8, 28].

The ideal purpose of a limb replantation of the upper extremity is to achieve a sensitive extremity with motor function which contains gripping, holding and supporting function. If these achievements seem to be unrealistic, the indication for a replantation should be made very critically in each individual case [13]. However, even in disastrous cases with additional avulsion and multilevel injuries the patient may benefit from a replantation even though it is from the very beginning evident that the functional outcome may be poor. Such an assessment can only be made in a center of replantation. Still, if a replantation is possible, it should be the primary approach. The patient and the family should be informed that in case of a loss of the replanted limb the challenge of an orthotropic cadaver transplantation is possible. Therefore a lifelong immunosuppression is mandatory with possible severe systemic consequences. Only few long-term results are available regarding the immunosuppressive effect in replanted limbs [29, 30]. The initial higher costs, caused by the complex and interdisciplinary treatment have to be confronted with the benefits of a possible reintegration into the work and social life. An unemployed and socially isolated patient can cause significantly higher long-term costs for society. Therefore, we also believe from an economic point of view, that the replantation even in disastrous cases seems to be favorable [13].

### Functional outcome

#### Upper arm

In patients with an amputation at the level of the upper arm securing the thoracohumeral pliers function, as well as active elbow flexion should be one of the principal achievements of therapy. Only the active elbow flexion allows the patient bimanual function of the hand.

#### Lower arm and wrist

At the forearm level the functional outcome depends mainly on the condition of the muscles in the proximal third and the quality of the reconstructed nerve [11]. The preservation of the wrist and adequate finger movement as well as the protective sensibility of the hand should be the principal achievements. According to the literature a functional limb can be restored in up to 41 % of the cases [11, 13]. In case of recovery of the grip function of the hand a power loss of up to 20–55 % compared to the contralateral non-injured side needs to be expected [27]. Often there is no regeneration of the intrinsic hand muscles, which may result in difficulties with fine mechanical work [27]. At this level of amputation about 20 % of the patients achieve a static two-point discrimination of <8 mm [27]. Regarding the functional results, a significant decline with increasing trauma, such as contusions or avulsions, multilevel injuries and age of the patient, must be expected. In addition to functional limitation such as ankylosis, capsulodesis, tenodesis, formation of a neuroma and lack of nerve regeneration may occur in over 20 %. In order to assess the function of the replanted limb German DASH questionnaire (Disability of Arm, Shoulder, Hand) can be used. The questionnaire measures the current subjective perception of the patient [31]. The patient himself assesses whether he meets the expected requirements according to rehabilitation and social reintegration. The assessment of functional impairment is calculated by the total score of the questions and the bandwidth of the questionnaire. A score of 0 represents a complete function without disability and a score of 100 an upper extremity without any function at all [31].

### Role of the age in macroreplantation

The functional outcome of a replantation of the upper limb is closely related to the age of the patient [14, 32].

The results of this study, using the DASH questionnaire, show that replantation of the upper limb were technically possible for the patients in the group >50 years, but the functional result turned out to be worse in the group >50 years than in the group of patients <50 years.

## Patients and methods

**Fig. 1:**
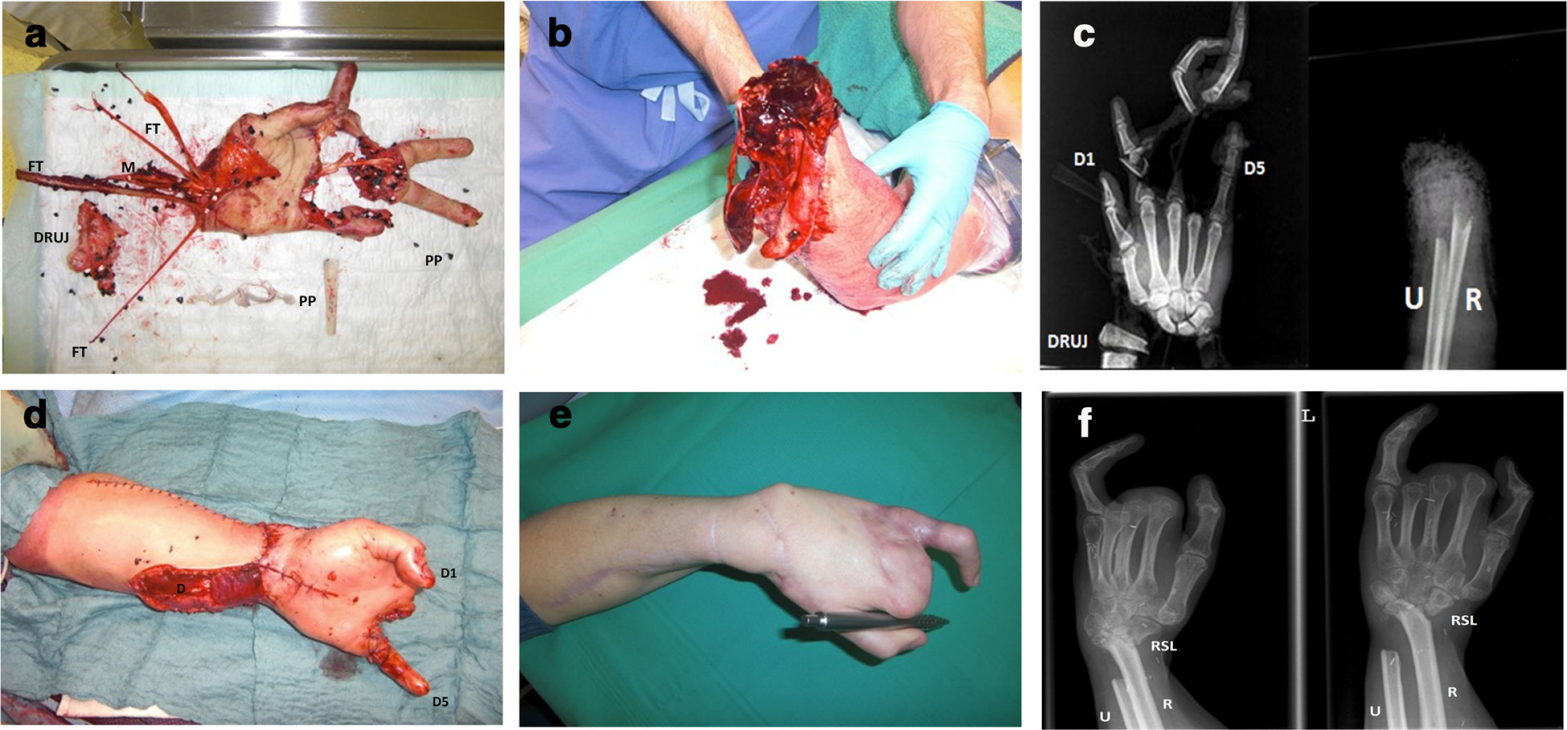
**a**: Amputated left hand of a 26 year old male at the level of the wrist preoperatively with a multilevel and avulsion injury through a plastic pellet machine. It shows additional subtotal amputation of the index finger and complete amputation of the middle- and ringfinger. The thumb was not injured; the little finger had a radial soft tissue defect. (PP) = black/white plastic pallets. (DRUJ) = distal radioular joint. (FT) = Flexor tendons. (M) = N. medianus. **b**: Preoperative situation of the stump of the distal forearm of the same 26 year old male. **c**: X-Rays of the left lower upper extremity and the left amputated amputated hand. (D1) = digit 1. (D5) = digit 5. The left picture shows a complete fracture of the distal radius and the distal ulna, with the distal radio ulnar joint lying transverse (DRUJ). The right picture shows the lower upper extremity with a fracture of the radius (R) and the ulna (U). **d**: Replanted hand postoperatively: thumb (D1) and little finger (D5) could be preserved, the index, middle and ringfinger were not re-plantable, the remaining defect (D) was temporarily covered by a negative pressure wound dressing and in a second operation 5 days later covered with split-skin-grafts. **e**: Replanted hand 9 months postoperatively: the patient is able to perform minimal flexion and adduction of the thumb in order to grip objects. **f**: X-Ray of the replanted hand 9 months postoperatively. (U) = ulna. (R) = radius. (RSL) = atypical RSL-arthrodesis of the left wrist

In the period from January 1^st^ 2003 and December 31^st^ 2011 six patients were replanted in our department after total or subtotal disastrous and complex macroamputation including additional injuries such as avulsion or multilevel injuries of the upper extremity. All patients have been treated by 4 senior plastic surgeons working in the same department with a standardized microsurgical treatment protocol. The only major changes within this period have been the implementation of new techniqual devices, e.g. new microscope or new microsurgical instruments. This retrospective clinical evaluation was approved by the local ethics committee of the University Hospital of Erlangen. The amputation was once at the level of the upper arm, once at the level of the elbow, three times the forearm was affected and once the wrist. All six patients were male. The average age was 49 years (25–73 years). Four patients had an accident at work, two of the patients were already pensioners who had an accident while performing private domestic work (Table 2). The accidents happened as followed: one patient had a smooth cut injury, one a convulsion injury, in three patients an avulsion injury was present with severe bruising and additional complex injuries of the amputated limb, one patient suffered a multilevel injury, a convulsion and avulsion injury of the forearm in addition to the amputation of three fingers (Fig. 1a–f).

**Table 2:** Overview of the 6 presented patients from 2003–2011

Patient	Sex	Age (years)	Level of Amputation	Industrial accident	Time of ischemia	Operating time (h)	Number of operations	Time of hospitalization (days)	Job before → after replantation
1	m	70	Elbow	n	03:29	01:55 h	16	59	Retired
2	m	25	Distal third of the upper arm	y	05:51	05:05 h	10	30	Truck driver → wholesaler
3	m	73	Wrist	n	05:46	04:58 h	4	31	Retired
4	m	26	Distal lower arm	y	06:17	08:20 h	5	17	Occupational retraining mechanic → Computer technician
5	m	40	Proximal lower arm	y	02:46	05:33 h	2	16	Metalworker, up-to-date: rehabilitation
6	m	62	Middle lower arm	y	04:13	04:52 h	3	23	Locksmith, retired earlier

## Results

Five of the six disastrous and complex macroamputations were replanted successfully. In one of the patients the replanted limb needed to be amputated at the level of the middle third of the forearm. The operation was followed by an implantation of a vascular AV-loop, which allowed covering the defect with a free rectus flap and splitting skin graft to obtain as much length of the stump as possible. None of the patients wanted a secondary amputation of the replanted limb. The time of cold ischemia was < 7 h in all patients. A stable plate fixation was applied with adequate soft tissue coverage in 2 of 6 cases. In 4 of 6 cases the fracture was treated with an external fixator. The patient who received a re-amputation later on, was initially scheduled for an arthrodesis of the elbow by external fixator. Two patients received a wrist arthrodesis primarily; one of them got in addition an arthrodesis of a metacarpo-trapezium joint. In 5 of these patients a debridement and shortening of the bone between 2–4 cm was necessary. In 3 of 6 patients vascular grafts were used. Three times the artery was reconstructed and four times the vein. In all patients, a residual soft tissue defect was initially covered temporarily with a negative pressure dressing. After the swelling significantly reduced the residual soft tissue defect could be covered with a split skin graft.

The mean average time of cold ischemia of the amputated limb, calculated from the time of the accident until the restoration of the arterial circulation, was 4:50 h (2:46 h–06:17 h). The operation lasted an average of 5:30 h (1:55–08:20 h). While the patients were hospitalized each of them received 7 operations (2–16) till the day of discharge. One patient required three days postoperatively a re-amputation of the replanted limb as well as a debridement of necrotic tissue due to vascular congestion. The operation was followed by several interventions including a vascular loop and later on a free rectus flap with split skin graft in order to cover the defect and keep the length of the limb. One patient received three days after the initial treatment a free latissimus dorsi flap to cover the soft tissue defect of the forearm. An opening of the compartment had to be conducted twice. During follow-up procedures eight re-osteosynthesis were necessary, 13 times a changing of the negative pressure dressing was required, seven split-thickness skin grafts were transplanted, one arteriolysis, one neurolysis, one arthrolysis, seven removals of osthesyntheses materials and one resection of a pseudarthrosis was conducted. In another patient a scar contracture of the elbow was resolved which required subsequently free anterolateral thigh flap 14 months after the trauma. The average time of hospitalization right after the trauma was 29 days (16–59 days).

All six patients received rehabilitation after dismissal. Two patients received inpatient rehabilitation for six weeks and received subsequently outpatient physical therapy and occupational therapy for 18 weeks. Another patient was four weeks in outpatient rehabilitation, followed by seven months of outpatient physical therapy and occupational therapy. Due to severe pain this patient needed to be treated four weeks in a hospital. One patient received outpatient physiotherapy exercising for nine months after discharge from the clinic. Two patients performed inpatient rehabilitation for 9 months.

The average follow-up time was 3.1 years (max 8 years min. 9 months). Two patients were already pensioners before the accident. Two patients had trained for a new job and are back to 100 % operational work. One patient was in the retraining for his former profession. One patient retired earlier.

Five of the six presented patients had completed the DASH questionnaire. For Part 1, in which the patient assesses the functioning of the replanted limb, the average of points was 71 (SD ± 13). For Part 2, in which the patient assesses the applicability of his replanted hand for work, sports and music the mean was 95 points (SD ± 10) and 96 points (SD ± 5).

All five patients were complaining about chronic pain in the area of replanted limb at time of the follow-up. Two of them mentioned moderate pain and three of them severe pain (Visual Analog Scale, VAS). Two stated that they regularly need pain medication; three said they take pain medication as needed.

All 6 treated patients mentioned that even looking back they had not preferred a primary stump, especially not the older patients with significantly higher functional limitations.

## Discussion

An amputation of the upper extremity and the following replantation is still one of the most challenging operations in the field of reconstructive surgery, especially in extremely severe cases of combined mutilating macroamputations including avulsion and multilevel injuries [8, 12]. Specialists agree that macroamputations with sharp wound edges are an absolute indication for replantation. However, there is no agreement in disastrous cases including avulsion and multilevel injuries. In these cases the chances for a good functional outcome are usually low as the outcome depends on several factors such as the type of accident and injury, the age and pre-existing disease of the patient, as well as time of ischemia and compliance. In all cases however, the reconstruction or the replantation of a limb is secondary in severely injured patients “at risk” e.g. for traumatic-hemorrhagic shock, severe brain injuries, chest trauma or other acute life threatening injuries. In these cases, “life before limb” is the most important aspect of the interdisciplinary treatment plan and a contraindication for lengthy replant or even reconstructive procedures [33].

The presented cases show, that even in severe and disastrous combined and complex macroamputations of the upper extremity, there may be an absolute indication for replantation. Even though the functional outcome may be poor due to the severe and complex injuries, most patients may benefit due to other e.g. psychological effects. Not only the feeling of physical integrity can be restored, but the replantation of an amputated upper extremity enables complete or partial recovery of function and sensibiliy of the arm which is important for the individual. Although our results show a very high DASH-Score, those achievements justify in several cases time and person consuming operations. Due to the retrospective study design and the treatment of 4 senior plastic surgeons on 6 patients, the presented data has a poor evidence level. However, we believe that every clinical data for this very small and severely injured patient group may help to reevaluate our interdisciplinary treatment strategies.

A replanted extremity is still superior to a secondary allotransplantation. Cases of an allogeneic transplantation of the upper extremity of a brain-dead donor have been published and discussed on a global basis. Worldwide 41 hands and two fingers in 32 patients were transplanted allogeneic between 1998 and 2008 [29, 30]. The time between amputation and transplantation varied from six months up to 35 years. Allogeneic limb transplantation needs to be regard as an organ transplant and therefore has immunogenicity [29, 30]. Therefore, several different acting immunosuppressive drugs must be taken a lifetime to prevent rejection. The immunosuppressive therapy in combination with antiviral and antibacterial drugs can lead to infection, arterial hypertension, diabetes mellitus, to liver and kidney failure, and an increased risk of developing certain types of cancer [30]. In two cases of the above mentioned 41 transplanted hands a secondary amputation was necessary. Therefore the indication for an allogeneic transplantation should be made very cautiously. The patient and his family must be informed in detail about the long intense time of recovery, as well as the risks and side effects of lifelong immunosuppressive therapy [30].

Even the use of prosthesis is in most cases not favored by the treated patients [13]. The technique of myoelectric prostheses is nowadays very advanced, so in case of a stump a prosthesis could be an alternative. With the help of electrodes, which detect the muscle activity in the stump, electric motors are driven, which can control the hand grip and rotation movements of the hand and the elbow. The disadvantages of prostheses are the heavy weight, and the higher price compared to the cosmetic prosthesis. An intense training can convert the prosthesis to a very useful tool in everyday life. In addition, the prosthesis is often superior to the replanted upper extremity from an aesthetic point of view. According to the literature the artificial replacement of the upper limb with a prosthesis is tolerated in only 20 to maximum 50 % of the cases [13].

Finally, despite the scientific achievements in the field of tissue engineering and experimental ex vivo perfusion of tissue at the present time there is no clinical applicability for replantation surgery [34–36]. Therefore, such severe injuries are best treated in the hands of experienced trauma and emergency surgeons with knowledge on the field of microsurgery [37].

## Conclusion

The presented cases show, that even in severe and disastrous combined and complex macroamputations of the upper extremity, there may be an absolute indication for replantation. The results of the DASH-Score in our patients are rather poor due to the severe and complex macroamputations including avulsion and multilevel injuries. Nevertheless all patients assure the usefulness of the replanted limb in their daily life. Although they cannot use the arm for very highly motoric movements, like playing an instrument with the hand and although they are highly limited with fine motoric movements in daily life, the limb seems be very helpful for them when it comes to gripping, holding and supporting function. The replanted limb gives them support in various daily requirements such as opening of doors. One patient mentioned that the limb is necessary for him to keep his body balance while he is walking or swimming.

The feeling of a still preserved sufficient physical integrity of a patient as well as the sensitive and significant functional advantages of replanted limb in comparison to dentures, justify the indication for a limb replantation with an enormous equipment and personal effort [10, 24]. After a realistic explanation of the functional prognosis of a formerly severely and complex injured replanted limb, the indication for replantation should not be limited by the age of the patient or the disastrous damage of tissue including avulsion and multilevel injuries, but by the patient’s individual needs and wishes and the individual motivation to restore the physical integrity.

### Statement

All three authors have read and complied with the instructions to authors and in particular the policy of our journal on ethical consent and standards of animal care.
